# Infection-Mediated Priming of Phagocytes Protects against Lethal Secondary *Aspergillus fumigatus* Challenge

**DOI:** 10.1371/journal.pone.0153829

**Published:** 2016-04-14

**Authors:** Amélie Savers, Orhan Rasid, Marianna Parlato, Matthias Brock, Gregory Jouvion, Bernhard Ryffel, Jean-Marc Cavaillon, Gerard Eberl, Oumaïma Ibrahim-Granet

**Affiliations:** 1 Institut Pasteur, Unité Cytokines & Inflammation, Paris, France; 2 Fungal Genetics and Biology, School of Life Sciences, University of Nottingham, Nottingham, United Kingdom; 3 INSERM UMR S1163 -Institut Imagine, Laboratoire d’Immunité Intestinale, Paris, France; 4 Institut Pasteur, Unité Histopathologie Humaine et Modèles Animaux, Paris, France; 5 *INSERM*, *UMR 7355*, Immunologie Moléculaire, Institut de Transgénose, Université d'Orléans et Centre National de la Recherche Scientifique, Orléans, France; 6 Institut Pasteur, Lymphoid Tissue Development Unit, Paris, France; Geisel School of Medicine at Dartmouth, UNITED STATES

## Abstract

Phagocytes restrict the germination of *Aspergillus fumigatus* conidia and prevent the establishment of invasive pulmonary aspergillosis in immunecompetent mice. Here we report that immunecompetent mice recovering from a primary *A*. *fumigatus* challenge are protected against a secondary lethal challenge. Using RAGγc knock-out mice we show that this protection is independent of T, B and NK cells. In protected mice, lung phagocytes are recruited more rapidly and are more efficient in conidial phagocytosis and killing. Protection was also associated with an enhanced expression of CXCR2 and Dectin-1 on bone marrow phagocytes. We also show that protective lung cytokine and chemokine responses are induced more rapidly and with enhanced dynamics in protected mice. Our findings support the hypothesis that following a first encounter with a non-lethal dose of *A*. *fumigatus* conidia, the innate immune system is primed and can mediate protection against a secondary lethal infection.

## Introduction

Over the past decades, *A*. *fumigatus* has emerged as an important opportunistic fungus that can cause severe human lung damage [1]. While healthy individuals rapidly clear inhaled conidia, immunecompromised patients have impaired clearance mechanisms that permit conidial germination in lung tissues, resulting in life-threatening invasive pulmonary aspergillosis.

Neutrophils, monocytes, and macrophages represent the first line of defense against fungal pathogens [2]. While alveolar macrophages have been shown to initiate the early inflammatory response [3], neutrophils are essential for fungal inactivation and, thus, protection against invasive aspergillosis [4] [5] [6].

Phagocyte expression of surface recognition receptors is essential for anti-fungal responses. The C-type lectin receptor Dectin-1 recognizes β-1,3-glucan and can initiate and mediate phagocytosis and pro-inflammatory cytokine responses [7]. Consitent with, Dectin-1 knock-out mice are more susceptible to *A*. *fumigatus* infection [8]. Beyond fungal recognition, an adequate immune response requires various cytokines, among these interleukin-17A (IL-17A) [9] [10]. IL-17 induces the expression of adhesion molecules on endothelial cells through p38-MAPK pathway, and increases the expression of chemokines like CXCL1, a ligand for CXCR2 [11]. Previously, it has been assumed that acquired immunity is exclusively mediated by T and B cells, however, innate immune cells such as NK cells have been shown to mediate immunological memory against viral infection [12]. Furthermore, “trained immunity” of monocytes following sub-lethal exposure to *Candida albicans* results in increased resistance to re-infection [13]. Furthermore, it has been shown that priming of mice by subcutaneous injection of heat-inactivated *A*. *fumigatus* germlings 3 days prior to infection reduces growth of *A*. *fumigatus* hyphae in a model of corneal stroma infection [9]. These latest studies indicate that, protection from fungal infections *via* priming of innate immune cells might, in principle, be possible. Therefore, by using an *in vivo* model of pulmonary aspergillosis we investigated the priming of innate immune cells and studied the resulting protection from otherwise lethal infectious doses of *A*. *fumigatus* conidia. To achieve this, we primed the immune system of mice by inhalation of a sublethal dose of viable *A*. *fumigatus* conidia. Subsequently, mice were infected with a lethal dose of conidia and the immune parameters of primed and naïve mice were compared with respect to disease progression and recovery, which were monitored by *in vivo* bioluminescence imaging [14]. We found that primed mice survived lethal challenge, and cleared the infection in 3 days. These results were also observed in RAGγc knock-out mice that are devoid of T, B and NK cells. We observed that primed mice have an elevated early pro-inflammatory cytokine production and are more effective at recruiting neutrophils to the infected airway. These cells have a primed phenotype (higher CXCR2 and Dectin-1 expression) and display heightened phagocytic and anti-fungal effector responses (more myeloperoxidase (MPO) and ROS). Interestingly, we found that bone marrow neutrophils of primed mice display these phenotypic changes, indicating a functional reprograming of phagocytic precursors. Our findings highlight an innate mechanism of immune protection to a secondary fungal challenge in the lung, in a clinically relevant setting of invasive aspergillosis, building on the developing concept of innate immune memory.

## Materials and Methods

### Fungal strain

The bioluminescent *A*. *fumigatus* strain Af 2/7/1 [14] was used in all experiments. This reporter strain derivative from the parental strains A1163 (CBS144.89) displays no virulence effects but allows monitoring of disease progression by bioluminescence imaging (BLI). Conidia from 8 days culture were harvested in phosphate-buffered saline (PBS) with 0.1% Tween20. For *in vivo* phagocytosis assays, conidia were stained with FITC as previously described [15].

### Ethics Statement

All procedures were carried out in accordance with Pasteur Institute guidelines in compliance with European guidelines. This study was approved by the ethical committee for animal experimentation CETEA (Comité d’éthique en expérimentation animale, Project license number 2013–0020).

### Laboratory inbred mice

RAG^*/-*^γc^*-/-*^ on B6 background mice were bred at the Pasteur Institute facilities. IL-17Ra^-/-^ mice were obtained from CDTA (Orleans CNRS, France). Wild-type BALB/c or C57BL/6 mice were obtained from the Centre d’ Elevage R. Janvier (France).

### Intranasal infection

Mice were infected with either a sub-lethal (5×10^7^ conidia; “SL”) or a lethal dose (5×10^8^ conidia; “L”) in a total volume of 25 μl. Mice within one group, named re-infected (“Re-Inf”), was given first the “SL” dose and 10 days later the “L” dose (termed day 0 in comparative analyses) (S1 Fig). All standard animal husbandry practices were followed during the course of study. Appropriate steps were adopted to keep the mice free from stress or discomfort. To further prevent distress to animals, humane endpoints were established at the very beginning of experiment. The measurement of body weight of the animals, was recorded daily and mice were euthanized when they lost 20% of their initial weight. In addition, throughout the study, the mice were examined 2 times daily for clinical signs such labored breathing, ruffled fur, hunched posture, impaired ambulation.for a period of 15 days. All surgery was performed under sodium pentobarbital anesthesia, and all efforts were made to minimize suffering.The animals were euthanized by CO_2_ asphyxiation at the end of the experiments.

### *In vivo* bioluminescence imaging

Images were acquired using the IVIS spectrum system (Perkin Elmer) as previously described [16]. In brief, mice were anesthetized using a constant flow of 2.5% isoflurane mixed with oxygen using an XGI-8 gas anesthesia system (Xenogen Corporation). 100 μl D-luciferin in PBS (33.3 mg/ml) was injected intra-peritoneally 10 min prior to photon detection. Data acquisition and quantification was performed using Living Image software version 3.1 (Xenogen Corporation). Quantification of photons per second was performed on defined regions of interest from the chest area.

### Collection of cells from broncho-alveolar lavage, blood and bone marrow

Broncho-alveolar Lavage (BAL) fluid was collected as previously described [15]. Blood cells were recovered by intracardiac puncture and bone marrow cells were collected by flushing the femoral shaft with 5 ml of PBS with a 23-gauge needle. Red blood cells were lysed using RBC lysis buffer (Multi-species; eBioscience) for 10 min at room temperature. Cell suspensions were washed twice and re-suspended in 1 ml of PBS.

### Antibodies and flow cytometry

Cells were incubated for 10 min in MACS buffer (PBS, 0.5% Fetal Calf Serum/FCS, 2 mM EDTA) containing 1 μg/ml of mouse Fc Block (Miltenyi Biotech) to prevent non-specific IgG binding to FcR-displaying leuKocytes. Cells were washed and stained for 30 min at 4°C. The following antibodies were purchased from BioLegend: GR-1 (APC or FITC), F4/80 (APC-Cy7), CD11a/CD18 (PerCP-Cy5) Dectin-1 (PE), CXCR2 (Alexa Fluor 647). The following antibodies were purchased from eBioscience: CD11b (PE-Cy7). Intracellular staining for MPO (PE) (Abcam) was performed on freshly sampled BAL cell suspensions. Cells were fixed and permeabilized using the Fixation & Permeabilization kit (eBiosciences). Reactive Oxygen species (ROS) from freshly isolated BAL neutrophils were quantified by flow cytometry using the ROS kit (Enzo). Data were acquired using a MACSQuant flow cytometer (Miltenyi Biotech). Samples were analyzed with MACSQuantify software (Miltenyi Biotech). All analysis was performed on total live cells in a sample.

### Phagocytosis assay of *A*. *fumigatus* conidia

Phagocytosis assays were performed as previously described [15]. In brief, mice were administered intranasally FITC-labeled conidia and BAL fluid was collected at different time points. Non-phagocytosed and surface attached conidia were distinguished from phagocytosed conidia by addition of an anti-conidia antibody that was detected using a secondary Texas Red-conjugated antibody. The phagocytosis rate was estimated as the ratio of the number of ingested conidia versus the total number of conidia associated with either 100 neutrophils or macrophages.

### Histological analysis of Lung sections

Lung sections were stained with Hemalun staining and methenamine silver for *A*. *fumigatus* detection and examined microscopically [14].

### Quantification of inflammatory mediators and colony forming units

Lung homogenates were obtained following disruption in saline using the Retsch Mixer Mill 301 homogenizer. IL-1α, IL-1β, TNFα, G-CSF, IL-6, CXCL1, and IL-17 concentrations in lung supernatants, BAL, and plasma were determined by ELISA as specified by the manufacturer (DuoSet; R&D Systems).

For assessment of fungal burden in mice, lung homogenates were serially diluted and plated on Malt Extract Agar supplemented with Chloramphenicol (0.5 mg/ml) at day 10 following infection with the sublethal dose.

### Statistical analyses

Results were statistically analyzed using GraphPad Prism 6.0 (GraphPad Software). The Mann Whitney test was used for two groups comparisons and the ANOVA two-way test with a Bonferroni post-test for multiple group comparisons. The Log-rank (Mantel-Cox) test was used for survival experiments. A *p* value below 0.05 was considered significant.

## Results

### A first sublethal challenge protects mice against a second lethal *A*. *fumigatus* dose

**Fig 1:**
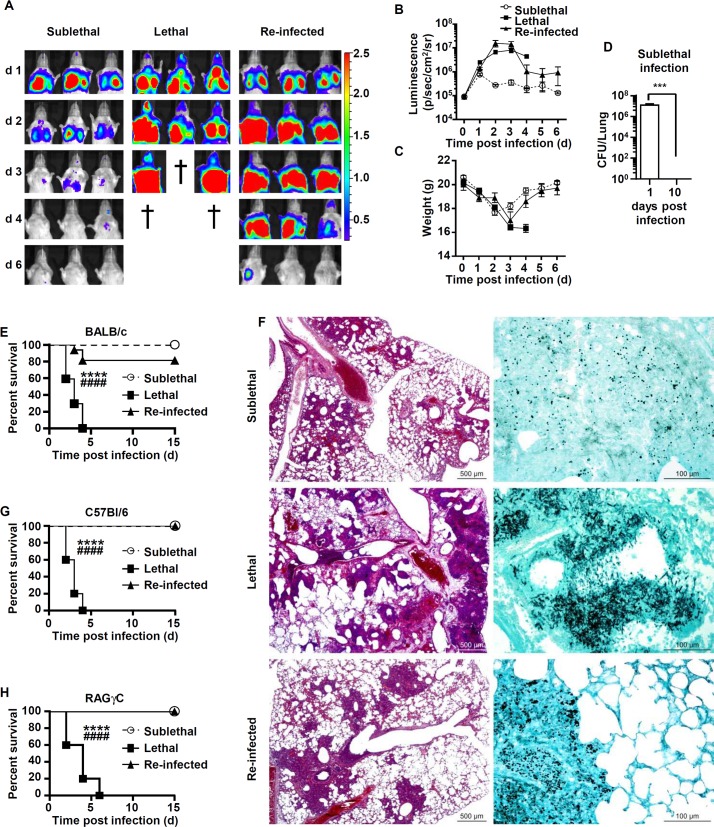
Mice recovering from sublethal *A*. *fumigatus* conidia inoculum are protected in a T-, B-lymphocytes or NK independent manner. Mice were either infected at day 0 with a sublethal (SL) or a lethal (L) concentration of *A*. *fumigatus* conidia. Re-infected (Re-Inf) mice were first infected with a SL dose and 10 days later were challenged with the L dose. The mice survival was followed on a daily basis during 15 days p.i. (A) *A*. *fumigatus-*emitted bioluminescence images from the thorax of representative BALB/c mice taken using the IVIS spectrum (Perkin) (B) Quantification (photons per second) of bioluminescence signal from mice chest areas using Living Image software (Perkin). (C) Modulation of the body weight in the three infection settings. (D) Colony forming unit from lungs homogenates. Mice were sacrificed from the “SL” group at day 1 and 10 and lung homogenates were collected and plated to determine the colony forming units (CFU). (E) Survival rate of BALB/c mice in the three infection settings. (F) Representative lung sections from mice at 48 h p.i. Left panels show Hemalun and right panel methenamine silver stained lung sections. Hemalun staining visualizes the inflammatory foci (purple), whereas silver staining visualizes fungal elements (black). (G) Survival rate of C57BL/6 mice in in the three conditions (H) Survival rate RAG^-/-^γc^-/-^mice in the three conditions. (****p = 0.0001 L dose *vs* SL dose; ####p<0.0001 L dose *vs* Re-Inf mice; n = 10 to 15 mice per group).

To study the effect of “priming” on murine survival following a secondary challenge with conidia, we first infected immunecompetent BALB/c mice with a sublethal dose (“SL”) of 5×10^7^ conidia of a bioluminescent *A*. *fumigatus* strain (S1 Fig). The SL mice experienced clinical disease characterized by weight loss and and we detected bioluminescence in the chest area for up to 3 days post-infection, followed by a decrease in the bioluminescence signal. The decrease in signal was and accompanied by weight gain starting at day 3 and a return to pre-challenge weight values by day 6, with no mortality observed (Fig 1A–1C and 1E). When we investigated lung histology on day 2 after challenge, we observed that neutrophils and macrophages formed inflammatory foci that were mostly centered on bronchioles, extended to alveoli, and contained very rare *A*. *fumigatus* hyphae (Fig 1F). On day 10, no luminescent signal or fungal growth was detected in the lungs.

The surviving mice from the “SL” group and control naïve mice were infected with a lethal dose of 5×10^8^ *A*. *fumigatus* conidia. (S1 Fig). These groups were annotated as “Re-Inf” and “L”, respectively. As expected, the “L” infected mice displayed severe disease progression, as assessed by increased bioluminescence signals and weight loss. In this group all mice succumbed to infection within 3 to 4 days (Fig 1E). Lung histopathology showed a typical necrotic pattern that was coupled with inflammatory foci in which cells were completely fragmented (karyorrhectic) and admixed in a granular acidophilic material that was rich in cell debris and red blood cells, indicative of vascular damage in the lung. Importantly, we observed a high density of *A*. *fumigatus* hyphae in the center of these lesions (Fig 1F).

Interestingly, re-infected mice “Re-Inf” that had controlled the primary “SL” infection exhibited a biphasic course of fungal disease. While an initial invasive fungal growth was observed, it did not progress beyond the first 3 days. The ensuing phase was characterized by fungal clearance and resulted in 80% survival of the “Re-Inf” BALB/c mice (Fig 1E). Furthermore, in this group, though the severity of inflammatory foci varied among different mice, a similar distribution of lesions was observed as for the lung sections of the “SL” mice (Fig 1F). Notably, the increased bioluminescence signal in the “Re-Inf” group was caused by the presence of *A*. *fumigatus* hyphae in lung sections of some mice (3/15) and was in agreement with the 20% of mice that succumbed to infection at day 3 to 4 post infection (p.i) (Fig 1E).

### Protection is independent of lymphocytes

To decipher the contribution of different cellular components of the immune system to the protective effect, we repeated our observations in C57BL/6 mice and performed comparative analyses between knock-out and wild-type mice in this background. Similar to the BALB/c background, C57BL/6 mice were protected in re-infection studies and displayed 100% survival while the “L” mice died within 4 days (Fig 1G). Subsequently, we analyzed a RAG^-/-^γc^-/-^ mouse strain that lacks all lymphocyte subsets. RAG^-/-^γc^-/-^ mice showed a very similar course of disease in the various groups and showed a similar protection pattern as the parental C57BL/6 mice (Fig 1H). These data indicate that protection in the “Re-Inf” group operates in a non T-, B—or NK-cell dependent manner.

### Phagocytes are rapidly recruited to the lungs in re-infected mice

**Fig 2:**
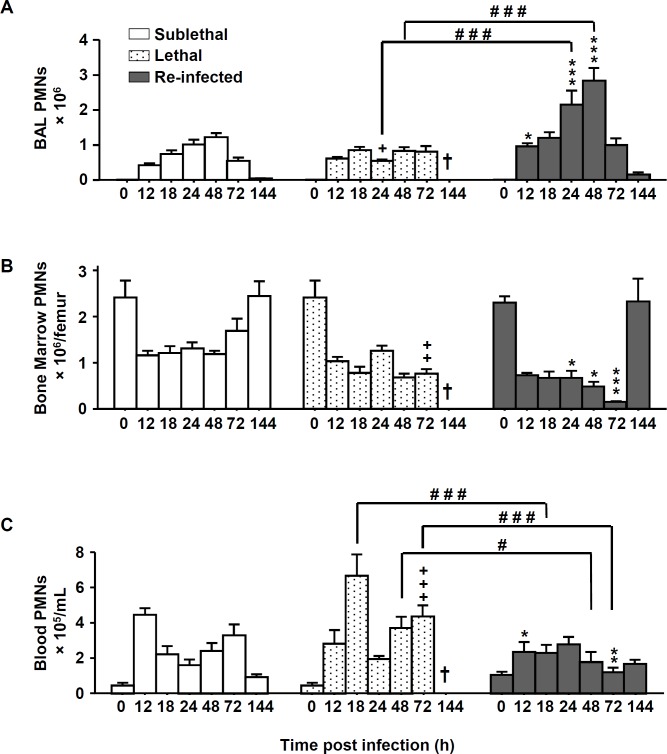
Neutrophils number is increased in re-infected protected mice. Mice were either infected at day 0 with a sublethal (SL) or a lethal (L) concentration of *A*. *fumigatus* conidia. Re-infected (Re-Inf) mice were first infected with a SL dose and 10 days later challenged with the L dose. Mice were sacrificed at 0, 12, 18, 24, 48, 72, and 144 h p.i. The time point 0 h p.i. of the Re-Inf mice corresponds to the day 10 of the SL mice (see S1 Fig). The BAL, bone marrow and blood cells were collected for flow cytometry analysis. Quantification of the total number of neutrophils (GR-1^+high^ CD11b^+^) from (A) BAL, (B) bone marrow and (C) blood during the course of infection. Data (mean ± SEM) represent 3 to 5 independent experiments with 5 mice per experiment. Statistically significant differences were determined using a Two way ANOVA with Bonferroni post-test (+p<0.05, ++p<0.01, +++p<0.001 L dose *vs* SL dose; *p<0.05, **p<0.01, ***p<0.001 Re-Inf *vs* SL dose; #p<0.05, ###p<0.001 LL dose *vs* Re-Inf mice).

Since the protective phenotype was independent of lymphocytes, we next analyzed the composition of phagocytic cells from bronchoalveolar lavages (BAL), blood and bone marrow by flow-cytometric analysis. Neutrophils were identified as Gr-1^high^ CD11b^+^ cells and the macrophages as F4/80^+^CD11b^+^GR-1^-^ cells (S2 Fig). Compared to the “SL” and “L” groups, the “Re-Inf” group showed a significantly increased number of neutrophils in the BAL at early time points. In this group the number of neutrophils peaked at 48h and then steadily declined, coincident with the reduction in the lung luminescent signal (Figs 2A and 1B). While the dynamics of neutrophil numbers in the bone marrow seemed to evolve similarly in the three groups, increased numbers of neutrophils were detected in the blood of lethal infected mice compared to the “Re-Inf” mice (Fig 2B and 2C). This observation is consistent with an impaired recruitment of these cells to the lung compartment (Fig 2A).

**Fig 3:**
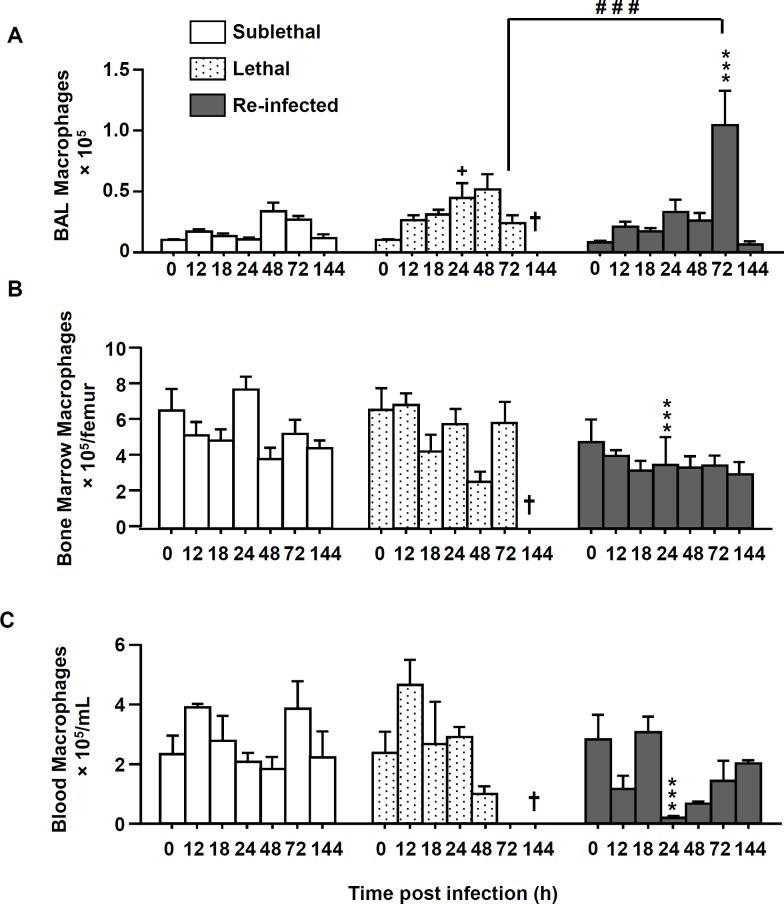
Macrophage numbers are increased in re-infected protected mice. Mice were either infected at day 0 with a sublethal (SL) or a lethal (L) concentration of *A*. *fumigatus* conidia. Re-infected (Re-Inf) mice were first infected with a SL dose and 10 days later were challenged with the L dose. Mice were sacrificed at 0, 12, 18, 24, 48, 72, and 144 h p.i. The time point 0 h p.i. of the Re-Inf mice corresponds to the day 10 of the SL mice (see S1 Fig). The BAL, bone marrow and blood cells were collected for flow cytometry analysis. Quantification of the total number of macrophages (F4/80+ GR-1^-^ CD11b^+^) from (A) BAL, (B) bone marrow and **(C)** blood during the course of infection. Data (mean ± SEM) represent 3 to 5 independent experiments with 5 mice per experiment. Statistically significant differences were determined using a Two way ANOVA with Bonferroni post-test (+p<0.05, ++p<0.01, +++p<0.001 L dose *vs* SL dose; *p<0.05, **p<0.01, ***p<0.001 Re-Inf *vs* SL dose; #p<0.05, ###p<0.001 LL dose *vs* Re-Inf mice).

In comparison to neutrophil recruitment patterns, the number of macrophages in the three compartments was generally lower than the number of neutrophils (Fig 3). Importantly, in the “Re Inf” group at 24 h pi, a significantly lower number of macrophages was observed in the bone marrow and blood compartments (Fig 3B and 3C). But interestingly, in this same group, in the BAL, the peak of recruited macrophages was observed at day 3 p.i. (Fig 3A) which coincides with the onset of fungal clearance (Fig 1C).

### Protection correlates with efficient phagocytosis and killing of conidia

**Fig 4:**
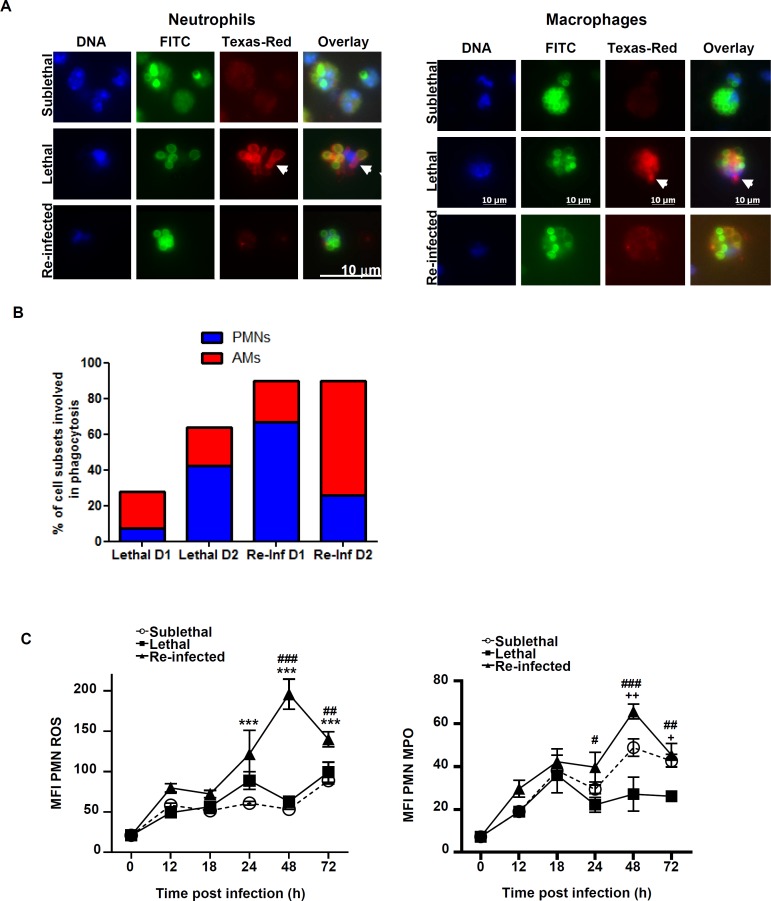
Phagocytes from protected mice internalize and kill the conidia efficiently. Mice were either infected at day 0 with a sublethal (SL) or a lethal (L) concentration of *A*. *fumigatus* conidia. Re-infected (Re-Inf) mice were first infected with a SL dose and 10 days later were challenged with the L dose. (See S1 Fig). (A) Representative pictures showing phagocytosis of conidia by BAL neutrophils (left panel) and macrophages (right panel) in the three infection settings at 48 h post infection. Mice were infected with FITC (green) labelled conidia, and the non-phagocytosed conidia were stained using an anti-conidia antibody detected by a secondary Texas Red-conjugated antibody (red). DNA was labelled with Hoechst stain (blue). Using fluorescence microscopy, the phagocytosed conidia were detected only in green while the outside FITC and Texas red conidia appear in yellow. Germination was indicated by the arrowheads pointing to non-phagocytozed and germinating conidia and to piercing hyphae (in red). The percentage of phagocytosis was estimated as the ratio of the number of ingested conidia to the total number of conidia bound to 100 phagocytes (B) Percentages of neutrophils (PMNs) and alveolar macrophages (AMs) involved in the phagocytosis in the lethal and re-infected groups at D1 and D2 h p.i. Within each histogram, is represented the percentage of either neutrophils or alveolar macrophages participating to the phagocytosis (C) BALs were collected and analyzed by flow cytometry to evaluate the killing potential. Plots are gated on GR1^high^ CD11b^+^ neutrophils and show expression level of the ROS (left panel) and MPO (right panel) production. Data (mean ± SEM) represent 2 to 3 independent experiments with 5 mice per experiment. Statistically significant differences were determined using a Two way ANOVA with Bonferroni post-test (+p<0.05, ++p<0.01, +++p<0.001 L dose *vs* SL dose; *p<0.05, **p<0.01, ***p<0.001 Re-Inf *vs* SL dose; #p<0.05, ###p<0.001 LL dose *vs* Re-Inf mice).

While phagocyte recruitment to the infected lung appeared more efficient in the “Re-Inf” group, we were also interested in the anti-fungal capacity of these cells. Phagocytosis and the killing of conidia is a critical step for survival [17]. Thus, BAL cells of “SL”, “L” and “Re-Inf” groups were analyzed for conidial phagocytosis of conidia at 24 and 48 h pi. As illustrated in Fig 4A, in the SL group 96% of the conidia were internalized by phagocytes 24 h pi. In agreement with lethal outcome in mice of the “L” group, only 28% of conidia in BAL samples were internalized by phagocytes at 24 h, a number that increased to 64% at 48 h. In contrast, 89% of conidia were already phagocytosed after 24 h in the “Re-Inf” mice and this number remained at 90% at 48h p.i. Thus conidial phagocytosis was more rapid in the “Re-Inf” group than in the”L” group.

Furthermore, the type of phagocytes involved in this process differed among the two groups. At 24 h in the “L” group, 73% of macrophages phagocytosed conidia while, at 48 h, 67% of neutrophils internalized the majority of conidia. In contrast, in the “Re-Inf” group neutrophils represented 75% of the cells that engulfed conidia at 24 h time point. This changed to 29% at 48 h with conidia primarily found in macrophages (71%) at this time point (Fig 4B). These data suggest that neutrophils conidial phagocytosis occurs more quickly and completely in the “Re-Inf” compared to the “L” group.

In the next experiments, we determined ROS and MPO production by neutrophils at various time points. Both neutrophil activity markers were significantly higher at 24 and 48 h in the “Re-Inf” group (Fig 4C). This coincides with an elevated number of neutrophils in the BAL of this group and implies that neutrophils actively contribute to the increased clearance and survival in the “Re-Inf” group.

### Protection correlates with early production of pro-inflammatory cytokines

**Fig 5:**
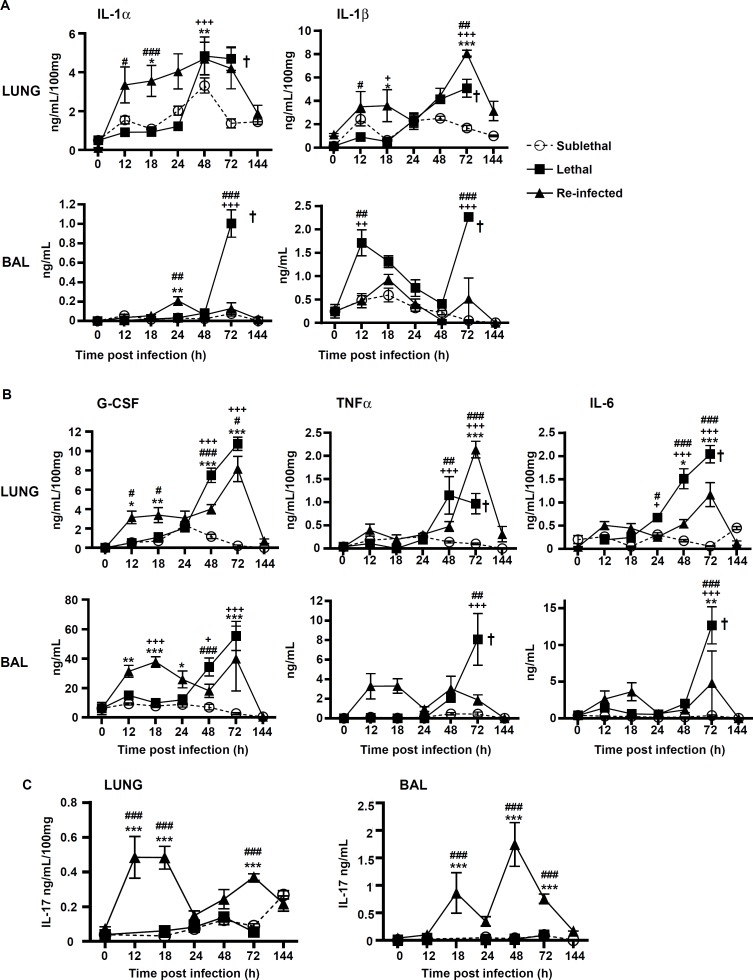
Protection correlates with early production of pro-inflammatory mediators. Mice were either infected at day 0 with a sublethal (SL) or a lethal (L) concentration of *A*. *fumigatus* conidia. Re-infected (Re-Inf) mice were first infected with a SL dose and 10 days later were challenged with the L dose. Mice were sacrificed at 0, 12, 18, 24, 48, 72, and 144 h p.i. The time point 0 h p.i. of the Re-Inf mice corresponds to the day 10 of the SL mice (see S1 Fig). The BAL and lungs tissue homogenates were were assessed by ELISA for quantification of (A) IL-1α, andIL-1ββ, (B) G-CSF, TNF α and IL-6, and (C) IL-17. Data (mean ± SEM) represent 2 to 3 independent experiments with n = 5 mice per experiment. Statistically significant differences were determined using a Two way ANOVA with Bonferroni post-test (+p<0.05, ++p<0.01, +++p<0.001 SL vs LL dose; *p<0.05, **p<0.01, ***p<0.001 Re-Inf vs SL dose; #p<0.05, ##p<0.01, ###p<0.001 LL dose vs Re-Inf mice).

We hypothesized that the pro-inflammatory response might be greater in “Re-Inf” mice compared to the other groups. Therefore, we studied the pro-inflammatory cytokines IL-1αIL-1β, G-CSF, TNFα, IL-6, in the lung tissue and BAL of all groups (Fig 5). In the lung tissues, the strongest early pro-inflammatory mediator profile was observed in the “Re-Inf” group, with a peak at 12–24 h (Fig 5A–5C). By comparison, the “L” group showed a delayed cytokine response that peaked at 48–72h in the lung and BAL. At this period, all animals suffered from severe acute infection and inflammation (Fig 5A and 5B) and a lethal outcome (Fig 1). In the “SL” group the weakest cytokine release was observed, coinciding with the lowest number of cells, inflammatory lesions and early control of conidia germination (Fig 5A–5C).

We also analyzed IL-17 production in lung and BAL in the different infection settings. In mice from the “SL” and “L” groups, IL-17 was only present at low levels during the time course of infection (Fig 5C). In contrast, the IL-17 profile dramatically differed in the “Re-Inf” group (Fig 5C), in which IL-17 was secreted in highest amounts in the BAL peaking at 18 and 48 h pi. This observation suggested an important role of IL-17 in the protection process.

To test this hypothesis, we evaluated the survival of IL-17 receptor A knock-out (IL-17RA^−/−^) mice. IL-17RA^−/−^ mice still have the ability to produce IL-17A, but signaling through its receptor IL-17RA is abrogated [18]. Our data showed that IL-17RA^-/-^ mice were partially susceptible to the sublethal dose of conidia with 71% survival. Following reinfection, only 30% survival was observed compared with 100% of the parental C57BL/6 mice (S3 Fig). To investigate the role of IL-17 signaling on neutrophils recruitment, we evaluated the number of BAL neutrophils 48 h following reinfection. Since the number of neutrophils in the BAL fluid of IL-17RA^-/-^ mice was three times lower than in wild-type mice (S3 Fig), not only production but also sensing of IL-17 appears to be important for the efficient recruitment of neutrophils.

### A low dose infection primes bone marrow phagocytes to increased Dectin-1 and CXCR2 expression

**Fig 6:**
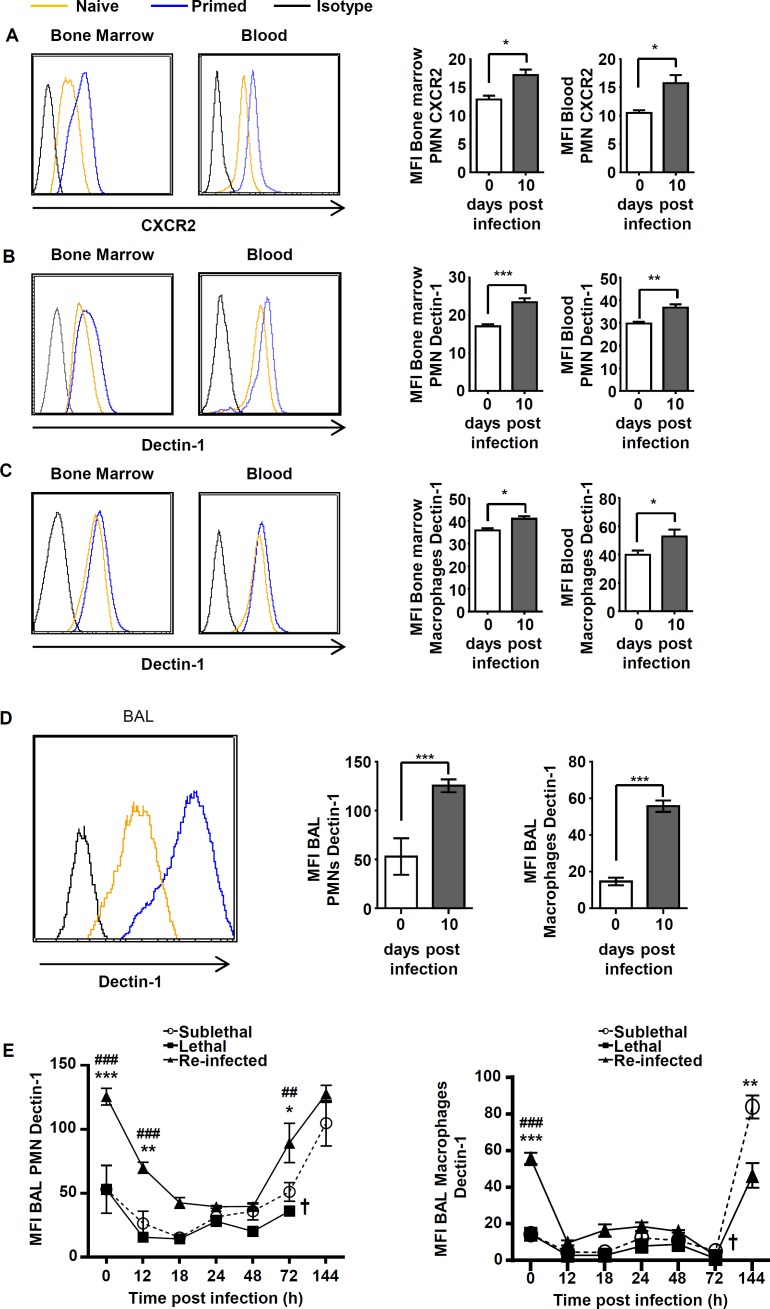
A sublethal infection primes bone marrow and blood phagocytes to increased Dectin-1 and CXCR2 expression. To investigate the priming process following the subethal infection, mice were either sacrificed at day 0 (Naïve) or at day 10 following infection with the sublethal dose (Primed). (A) CXCR2 expression on bone marrow and blood neutrophils (GR1^high^ CD11b^+^ population). Dectin-1 expression levels on bone marrow and blood neutrophils (GR1^high^ CD11b^+^ population) (B), and macrophages (F4/80^+^CD11b^+^GR-1^-^ population) (C). (D) Dectin-1 expression levels on BAL neutrophils and macrophages. Representative histograms show level of expression (left panel) and quantification of respective values in graphs (right panel). (E) Dynamic of Dectin-1 level expression on both neutrophils (left panel) and macrophages (right panel) in the BALs in the three infection settings. “SL” or “L” mice were sacrificed at 0 h (naïve), 12 18, 24, 48, 72 and 144 h p.i. and “Re-inf” mice were sacrificed at 0 h (10 days after the SL infection, day of the re-infection), 12, 18,24, 48, 72 and 144 h p.i. Data (mean ± SEM) represent 2 to 3 independent experiment with n = 5 mice per experiment. Statistically significant differences were determined using a Two way ANOVA with Bonferroni post-test (+p<0.05, ++p<0.01; *p<0.05, **p<0.01, ***p<0.001Re-Inf *vs* SL dose; #p<0.05, ##p<0.005 ###p<0.001 LL dose *vs* Re-Inf mice).

Phagocytes were most rapidly recruited in “Re-Inf” mice. Due to the importance of CXCR2 signaling in efficient neutrophil recruitment and host defense [4] [19] [5] [20], we tested CXCR2 and Dectin-1 expression on cell subsets within the BM, blood and BALs in the three experimental settings. We found that both, CXCR2 and Dectin-1 were expressed at significantly elevated levels in bone marrow and blood phagocytes at day 10 of”SL” mice i.e. primed mice (Fig 6A–6C). Dectin-1 was also expressed at significantly higher levels in BAL neutrophils and macrophages on day 10 in SL mice (Fig 6D). In agreement with its receptor function, Dectin-1 expression on BAL phagocytes significantly decreased during the early time course of infection, but always remained highest in the “Re-Inf” group (Fig 6D and 6E). At time points of clearance of the infection the Dectin-1 levels re-appeared, whereby a slight delay was observed with macrophages (Fig 6E). This is in agreement with a switch from neutrophil to macrophage phagocytosis as described. The high Dectin-1 levels in the “SL” group on both types of phagocytes at 144 h post infection remained at this high level and appear to be the result of the early modulation of receptor expression in the bone marrow of primed mice. This is supported by the fact that high Dectin-1 levels were maintained at least until day ten in the “SL” group (day of re-infection), although fungal elements from the first infection were no longer observed and no colony forming units derived from lung homogenates of “SL” mice at day 10.

In conclusion, these results suggest that due to elevated CXCR2 expression phagocytes and especially neutrophils are more rapidly recruited to the site of infection, where they are able to phagocytose more efficiently thanks to an increased expression of Dectin-1.

## Discussion

Previous studies have shown that the innate immune system can be trained to recognize fungal infections. Examples are the “trained immunity” of monocytes following sublethal exposure to *Candida albicans* [13] or the control of growth of *A*. *fumigatus* hyphae in a model of corneal stroma infection after subcutaneous injection of heat-inactivated *A*. *fumigatus* germlings [9]. While the latter study used heat inactivated conidia, we were interested in the power of challenging mice with live conidia with a sublethal dose. It is worth noting, that based on the high resistance of immunecomptent mice, a high fungal burden was required to generate an experimental pathogenicity weakening the translational relevance of our model.

With this setting we investigated the protection against a subsequently applied life-threatening challenge with live conidia. In addition, we were interested in the recruitment and action of phagocytes, the inflammatory response and surface receptor expression.

An important observation derived from the fact that protection occurred even in the absence of lymphocytes, since RAG^-/-^γc^-/-^ mice that lack adaptive immune cells and NK cells were protected. This confirms that homologous protection is derived from non-lymphoid cells. Phagocytes were recruited in much higher number and much more rapidly when mice had been primed with a sublethal dose of live conidia. We wondered by which mechanism the recruitment velocity of neutrophils had increased. Analyses of bone marrow phagocytes revealed increased expression of CXCR2 and Dectin-1. Although additional receptors may be upregulated, it confirms that at least one important receptor for recruitment (CXCR2) and one receptor for recognition of fungal elements (Dectin-1) are expressed at higher levels in relevant phagocyte populations in the bone marrow. This indicates that a re-programming had occurred that adapts phagocytes towards a fungal infection. However, further investigations will be required to answer this very important but controversial question in more detail.

Interestingly, we also detected a significantly elevated level of IL-17 in protected mice. Its production and sensing is an essential contributor to resistance [9]. Although 71% of IL-17RA^-/-^ mice survived the “SL” infection, they largely succumbed to re-infection. This was accompanied by a reduced neutrophil influx and indicates that both, IL-17 production and sensing contribute to the recruitment process.

In conclusion, we assume that the priming process is initiated in the bone marrow, resulting in phagocytes with high Dectin-1 and CXCR2 expression. Efficient phagocytosis and increased ROS and MPO production allows efficient control of fungal germination. Thus, our model extends the fundamental understanding of the role of phagocytes in the innate immunity against aspergillosis, and suggests a memory mechanism for increased reactivity against a second encounter with fungal pathogens. Detailed analysis of chemokine and cytokine production, phagocyte influx, phagocytosis, and killing capacity uncovered a cascade of events that ultimately leads to protection.

## Supporting Information

S1 Fig Infection strategy. Mice were either infected at day 0 with a sublethal “SL” or a lethal “L” concentration of *A*. *fumigatus* conidia. Re-infected “Re-Inf” mice were first infected with a SL dose and 10 days later were challenged with the L dose. The mice survival was followed on a daily basis during 15 days p.i. To investigate the cell recruitment, the inflammatory response and the expression of CXCR2 and Dectin-1 mice were sacrificed at different times as indicated.
(DOCX)

S2 Fig Cytometry Gating strategy. Mice were infected and the different cell populations recovered at day 2 following infection. Representative flow cytometry analysis with plots from bone marrow, blood and BAL showing GR-1^+high^ CD11b^+^ neutrophil staining (upper panel) and F4/80^+^CD11b^+^GR-1^-^macrophages staining (lower panel) The percentages of the neutrophils and the macrophages are indicated in the gates.
(DOCX)

S3 Fig IL-17 Receptor is important for neutrophils release and protection. Wild type and IL-17 RA -/- were infected with the sublethal inoculum and survival followed for 15 days (A upper panel). Lower panel shows the survival of WT versus IL-17 RA -/- reinfected mice following 10 days infection with the sublethal dose. Survival rate (####p<0.0001) between WT and IL-17 RA -/- mice was given following a Kaplan-Meier log-rank test. (B) Representative flow cytometry plots showing GR-1^+high^ CD11b^+^ neutrophils staining at 48 h post re-infection of wild type and 17 RA -/- mice.(Upper panel). Percentages represent the upper right quadrant. In the lower panel is shown the quantification of the total number of neutrophils GR1high CD11b+.
(DOCX)
