# Supplementary material for: Infection-Mediated Priming of Phagocytes Protects against Lethal Secondary Aspergillus fumigatus Challenge
Source: PLoS One. 2016 Apr 14;11(4):e0153829. doi: 10.1371/journal.pone.0153829 (PMC4831689; doi:10.1371/journal.pone.0153829)

**S1 Fig**. **Infection strategy**

Mice were either infected at day 0 with a sublethal “SL” or a lethal “L” concentration of *A. fumigatus* conidia. Re-infected “Re-Inf” mice were first infected with a SL dose and 10 days later were challenged with the L dose. The mice survival was followed on a daily basis during 15 days p.i. To investigate the cell recruitment, the inflammatory response and the expression of CXCR2 and Dectin-1 mice were sacrificed at different times as indicated.


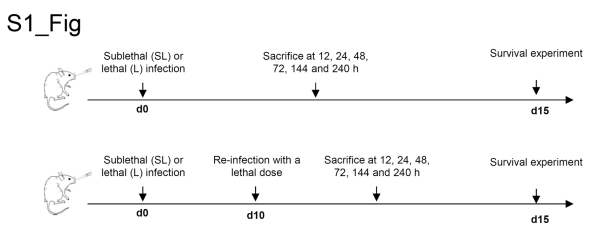

Supplement: S1 Fig — Mice were either infected at day 0 with a sublethal “SL” or a lethal “L” concentration of A. fumigatus conidia. Re-infected “Re-Inf” mice were first infected with a SL dose and 10 days later were challenged with the L dose. The mice survival was followed on a daily basis during 15 days p.i. To investigate the cell recruitment, the inflammatory response and the expression of CXCR2 and Dectin-1 mice were sacrificed at different times as indicated. (DOCX) [file pone.0153829.s001.docx]
