# Supplementary material for: Infection-Mediated Priming of Phagocytes Protects against Lethal Secondary Aspergillus fumigatus Challenge
Source: PLoS One. 2016 Apr 14;11(4):e0153829. doi: 10.1371/journal.pone.0153829 (PMC4831689; doi:10.1371/journal.pone.0153829)

**S2 Fig Cytometry Gating strategy**

Mice were infected and the different cell populations recovered at day 2 following infection. Representative flow cytometry analysis with plots from bone marrow, blood and BAL showing GR-1^+high^ CD11b^+^ neutrophil staining (upper panel) and F4/80^+^CD11b^+^GR-1^-^macrophages staining (lower panel) The percentages of the neutrophils and the macrophages are indicated in the gates.


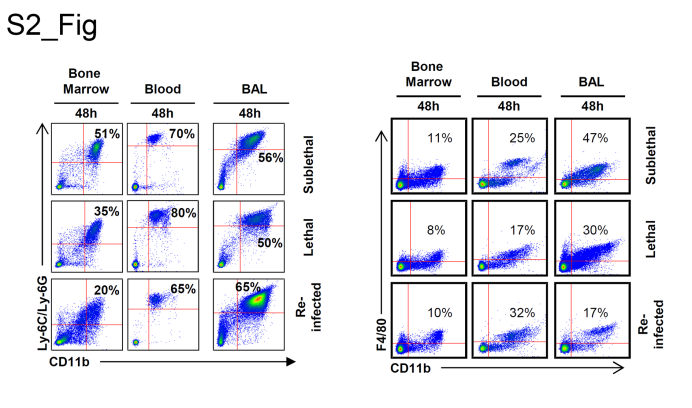

Supplement: S2 Fig — Mice were infected and the different cell populations recovered at day 2 following infection. Representative flow cytometry analysis with plots from bone marrow, blood and BAL showing GR-1+high CD11b+ neutrophil staining (upper panel) and F4/80+CD11b+GR-1-macrophages staining (lower panel) The percentages of the neutrophils and the macrophages are indicated in the gates. (DOCX) [file pone.0153829.s002.docx]
