# Supplementary material for: Infection-Mediated Priming of Phagocytes Protects against Lethal Secondary Aspergillus fumigatus Challenge
Source: PLoS One. 2016 Apr 14;11(4):e0153829. doi: 10.1371/journal.pone.0153829 (PMC4831689; doi:10.1371/journal.pone.0153829)

**S3 Fig IL-17** **Receptor is important for neutrophils release and protection**.

Wild type and IL-17 RA -/- were infected with the sublethal inoculum and survival followed for 15 days (A upper panel). Lower panel shows the survival of WT versus IL-17 RA -/- reinfected mice following 10 days infection with the sublethal dose. Survival rate (####p<0.0001) between WT and IL-17 RA -/- mice was given following a Kaplan-Meier log-rank test. (B) Representative flow cytometry plots showing GR-1^+high^ CD11b^+^ neutrophils staining at 48 h post re-infection of wild type and 17 RA -/- mice .(Upper panel). Percentages represent the upper right quadrant. In the lower panel is shown the quantification of the total number of neutrophils GR1high CD11b+.


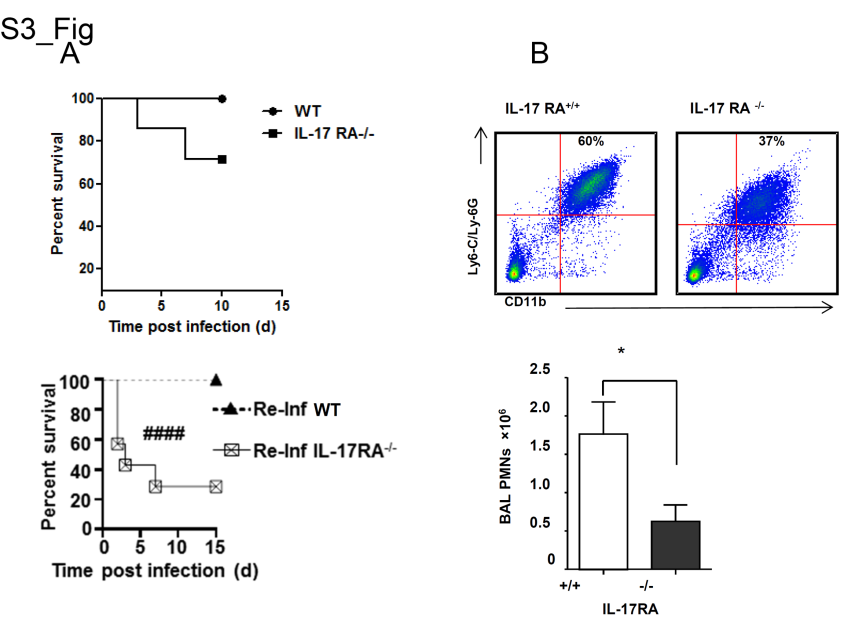

Supplement: S3 Fig — Wild type and IL-17 RA -/- were infected with the sublethal inoculum and survival followed for 15 days (A upper panel). Lower panel shows the survival of WT versus IL-17 RA -/- reinfected mice following 10 days infection with the sublethal dose. Survival rate (####p<0.0001) between WT and IL-17 RA -/- mice was given following a Kaplan-Meier log-rank test. (B) Representative flow cytometry plots showing GR-1+high CD11b+ neutrophils staining at 48 h post re-infection of wild type and 17 RA -/- mice.(Upper panel). Percentages represent the upper right quadrant. In the lower panel is shown the quantification of the total number of neutrophils GR1high CD11b+. (DOCX) [file pone.0153829.s003.docx]
